# Interstitial lung abnormalities: to treat or not to treat? The hamlet dilemma

**DOI:** 10.3389/fmed.2026.1762243

**Published:** 2026-02-13

**Authors:** Umberto Zanini, Giovanni Franco, Chiara Pirotta, Eleonora Passeri, Sofia Maria Mazzotta, Giovanni Ferrara, Marco Mura, Paola Faverio, Fabrizio Luppi

**Affiliations:** 1 Fondazione IRCCS San Gerardo dei Tintori, SC Pneumologia, University of Milano Bicocca, School of Medicine and Surgery, Monza, Italy; 2 Division of Pulmonary Medicine, Faculty of Medicine and Dentistry, University of Alberta, Edmonton, AB, Canada; 3 Department of Pathology and Laboratory Medicine, Western University, London, ON, Canada

**Keywords:** early treatment, interstitial lung abnormalities, monitoring, pulmonary fibrosis, risk assessment

## Abstract

Interstitial lung abnormalities (ILAs) are increasingly recognized as incidental findings on chest computed tomography and may represent an early stage in the spectrum of interstitial lung disease (ILD). However, their clinical significance and optimal management remain debated. While some ILAs may progress to pulmonary fibrosis and functional impairment, many remain stable, and the criteria for initiating diagnostic evaluation, determining monitoring intensity, and starting therapeutic interventions are not clearly established. Current guidelines emphasize risk stratification and longitudinal surveillance, yet significant uncertainties persist regarding which individuals may benefit from early treatment rather than conservative follow-up. This narrative review synthesizes the existing evidence on ILA epidemiology, natural history, and proposed management approaches. We analyse data supporting both early intervention—particularly when ILAs evolve into clinically meaningful ILD—and watchful waiting strategies to avoid overtreatment in asymptomatic and stable cases. The review highlights persistent knowledge gaps, including the lack of consensus on defining progression, determining optimal follow-up intervals, and identifying clear indications for pharmacologic therapy. Overall, ILAs represent a heterogeneous and still poorly defined entity. By critically examining current evidence and ongoing debates, this review aims to guide clinicians in navigating the “to treat or not to treat” dilemma and to outline key priorities for future research.

## 1 Introduction

Interstitial lung abnormalities (ILA) are incidental findings on chest computed tomography (CT) that may indicate underlying interstitial lung disease (ILD) or can represent an early stage of pulmonary fibrosis (1, 2).

In 2020, the Fleischner Society proposed a first standardized definition for ILA—later refined in a 2025 American Thoracic Society statement—characterizing them as bilateral, non-dependent parenchymal abnormalities involving at least 5% of any lung zone, including ground-glass opacities, reticular abnormalities, parenchymal distortion, traction bronchiectasis, or honeycombing (1, 2).

ILA is uncommon in those under 50, with a prevalence of about 7% in the general adult population and up to 20% among smokers or lung cancer screening cohorts (3–5). Other risk factors include older age, male sex, a family history of pulmonary fibrosis, and genetic variants (1, 6). Additional risks involve exposure to inhaled substances, connective tissue diseases, and reduced forced vital capacity (FVC) (1, 7, 8).

ILAs are imaging-based features that do not necessarily correspond to clinical symptoms or functional impairment, and their natural history is heterogeneous (1, 2). They are frequently detected incidentally on chest CT in individuals without symptoms or clinical suspicion of ILD, but longitudinal studies have demonstrated that a substantial proportion of ILAs—particularly those with subpleural fibrotic features—progress over time to overt ILD, with associated declines in lung function and exercise capacity (1, 2).

Given the heterogeneous natural history and limited understanding of which individuals will progress to clinically significant disease, the optimal management of ILA remains uncertain, highlighting the need for evidence-based strategies to guide surveillance and potential therapeutic interventions. Our review aims to underline the current evidence regarding the criteria for treating and not treating ILAs.

## 2 Materials and methods

A search of relevant medical literature in the English language was conducted for this narrative review in Medline/PubMed, EMBASE and Scopus for articles published up to August 2025, including observational, interventional studies, reviews and guidelines. Keywords used to perform the research are reported in Table 1. Editorials, conference abstracts, case stories, smaller case series and pre-print publications were excluded. Relevant abstracts and articles were searched and screened independently by 3 authors (UZ, GF and SMM). In cases of disagreement, the articles were collectively reviewed, considering their relevance, strengths, and limitations. Articles in other languages with abstract in English were also reviewed if sufficient detail was present in the abstract.

**Table 1:** Keywords used to perform the research.

Topic	Search string (PubMed)
Definition and epidemiology	(“interstitial lung abnormalities” OR “ILA”) AND (“definition” OR “classification” OR “Fleischner Society” OR “ATS statement” OR “epidemiology” OR “prevalence”)
Radiologic and clinical predictors of progression	(“interstitial lung abnormalities” OR “ILA”) AND (“progression” OR “fibrosis progression” OR “radiologic progression” OR “lung function decline” OR “traction bronchiectasis” OR “honeycombing” OR “subpleural fibrotic”)
Genetic and molecular biomarkers	(“interstitial lung abnormalities” OR “ILA”) AND (“biomarkers” OR “MUC5B” OR “telomere length” OR “KL-6” OR “surfactant protein-D” OR “matrix metalloproteinases” OR “SP-D”)
Therapeutic approaches and antifibrotic therapy	(“interstitial lung abnormalities” OR “ILA”) AND (“antifibrotic therapy” OR “nintedanib” OR “pirfenidone” OR “treatment” OR “early intervention” OR “progressive fibrosing interstitial lung disease”)
Monitoring and follow-up strategies	(“interstitial lung abnormalities” OR “ILA”) AND (“follow-up” OR “surveillance” OR “monitoring” OR “management” OR “risk stratification” OR “multidisciplinary discussion”)

## 3 Radiologic, clinical, and molecular determinants of progression in ILAs

Differentiating ILAs from clinically overt ILD is essential for effective management. The Fleischner Society and the American Thoracic Society (ATS) documents indicate that ILAs should not be classified as clinically significant ILD, but rather as an at-risk group that requires monitoring and risk stratification (1, 2). The distinction between ILAs and other ILDs is crucial because it affects access to antifibrotic or immunomodulatory therapies, provided to that patients who meet the criteria for a defined interstitial lung disease receiving appropriate care and do not undergo overtreatment for true ILAs. Reported rates of ILA progression vary widely across studies, ranging from approximately 20% to over 80% (4, 9–14). This heterogeneity reflects differences in study populations, imaging definitions, follow-up duration, and criteria used to define progression. Population-based and lung cancer screening cohorts generally report progression rates of 20–76% over 2–12 years, with higher rates in individuals showing fibrotic patterns, older age, smoking history, or genetic susceptibility (e.g., MUC5B variant). Additional variability arises from divergent imaging assessment methods—visual versus quantitative—and inconsistent inclusion of fibrotic and non-fibrotic subtypes. Meta-analyses highlight that these methodological differences, combined with variable risk stratification approaches, limit comparability across studies (5).

On chest CT, ILAs are classified into three patterns based on their distribution and the presence of fibrotic changes: non-subpleural, subpleural non-fibrotic, and subpleural fibrotic (1, 2). This classification is clinically relevant reflecting different risks of progression toward established ILD. In particular, subpleural fibrotic ILA represents the subtype most strongly associated with disease progression and adverse outcomes (15, 16). Radiologic predictors of progression include the presence and extent of fibrotic changes (such as traction bronchiectasis, bronchiolectasis, or honeycombing), as well as a subpleural and basal predominance of abnormalities (17). Specifically, subpleural fibrotic ILA is associated with a markedly higher risk of progression (hazard ratio up to 8.4) and mortality compared with non-fibrotic ILA (18). According to the ATS guidelines, fibrotic features resembling usual interstitial pneumonia (UIP) confer the highest risk for adverse outcomes (1). Moreover, quantitative assessment of fibrosis extent is independently associated with disease evolution: a fibrotic burden of ≥1% of total lung or ≥5% of a lung zone, and the presence of honeycombing, are linked to greater radiologic progression and poorer long-term survival (1, 14). Extensive reticulation in subpleural non-fibrotic ILA also predicts progression, with risk levels approaching those of fibrotic ILA, whereas non-subpleural ILAs are generally stable and not associated with increased mortality (2).

Another important issue in evaluating ILAs is determining whether the findings are longstanding or have developed recently. This distinction has a significant impact on clinical interpretation and management. Reviewing previous chest imaging, such as earlier chest X-rays or abdominal CT scans that include the lung bases, can help establish the chronicity of the findings. Stable interstitial changes over time may indicate residual scarring or non-progressive abnormalities. In contrast, newly detected ILAs, especially those exhibiting fibrotic features, require closer monitoring and further assessment due to their higher likelihood of progression.

Clinical key risk factors suggested for ILA progression include advanced age, smoking, environmental or occupational exposures. In fact, recent clinical statements from the American Thoracic Society and systematic reviews have consistently identified older age as a significant risk factor for both the presence and progression of ILAs (1). Meta-analyses and cohort studies further confirm that age remains a robust predictor of both ILA development and adverse outcomes, independent of other risk factors (5). However, the commonly used age threshold of over 50 years should be interpreted with caution (4). Since ILAs are rarely found in younger individuals, this cutoff may reflect the age distribution of populations undergoing chest imaging rather than a true biological threshold. Therefore, age is better understood as a continuous risk gradient rather than a discrete categorical variable.

Moreover, smoking and exposure to environmental and occupational factors are well-established risk factors for the development and progression of ILAs (19). Beyond its association with smoking-related interstitial lung diseases, such as respiratory bronchiolitis–ILD and desquamative interstitial pneumonia, cigarette smoking has been consistently associated with an increased prevalence and progression of ILAs in the general population (20). Both smoking intensity and current smoking status correlate with fibrotic patterns, accelerated decline in lung function, and higher mortality, supporting the hypothesis that smoking may contribute to the transition from subclinical interstitial abnormalities to clinically significant fibrotic ILD (21). Similarly, exposure to air pollution, mold, dust, fumes, and occupational agents independently increases the risk of ILA presence and progression, particularly in genetically susceptible individuals (7, 22). Comprehensive assessment of smoking history and environmental or occupational exposures is therefore essential for identifying patients at higher risk and guiding monitoring and preventive strategies.

Autoimmune mechanisms may contribute to the development and progression of interstitial lung abnormalities (ILAs), where ILAs may represent an early or subclinical manifestation of CTD-associated interstitial lung disease (CTD-ILD) (23, 24). The prevalence of radiologic interstitial lung involvement, encompassing both subclinical ILAs and overt ILD, is high in connective tissue disease populations, affecting approximately 40% of patients with high-risk CTDs (25).

ILAs identified on high-resolution CT scan precede the onset of respiratory symptoms or serologic evidence of CTD by several years, supporting their role as an early imaging marker along the CTD-ILD disease spectrum (26).

In fact, in genetically predisposed individuals, aberrant immune activation and autoantibody formation—such as anti-nuclear, anti-citrullinated peptide, or myositis-specific antibodies—may trigger persistent alveolar inflammation and fibrotic remodeling through cytokine-mediated pathways (23, 24).

The presence of autoantibodies or subtle extrapulmonary autoimmune features has been associated with an increased risk of progression from subclinical interstitial abnormalities to clinically overt CTD-ILD (27, 28).

Accordingly, the detection of ILAs in patients with established CTD or in individuals at risk for autoimmune disease should prompt a careful evaluation for underlying CTD and justify longitudinal clinical, functional, and radiologic monitoring, as early ILAs may evolve into clinically significant interstitial lung disease with important prognostic and therapeutic implications (29).

ILAs are increasingly identified in patients with cancer, largely due to the widespread use of chest computed tomography for staging, screening, and treatment monitoring. In this setting, ILAs may reflect pre-existing subclinical interstitial lung disease or early fibrotic changes that interact with cancer-related factors and oncologic treatments. A recent meta-analysis in lung cancer patients reported a pooled ILA prevalence of approximately 9–17% and demonstrated that baseline ILAs are associated with worse overall survival and an increased risk of treatment-related pulmonary toxicity (30).

Growing evidence indicates that ILAs represent a relevant risk factor for pulmonary complications during cancer therapy, particularly in patients receiving immune checkpoint inhibitors, chemotherapy, or thoracic radiotherapy. Several studies have shown that pre-existing ILAs—especially subpleural fibrotic patterns—are associated with a substantially higher incidence and severity of immune checkpoint inhibitor–related pneumonitis, as well as poorer oncologic outcomes (31). Similarly, in patients undergoing definitive chemoradiotherapy for locally advanced lung cancer, fibrotic ILAs have been independently associated with an increased risk of symptomatic radiation pneumonitis and reduced survival (32).

Accordingly, ILAs detected in patients undergoing oncologic therapies should be regarded as a high-risk clinical feature. Their presence warrants individualized management, closer respiratory surveillance, and multidisciplinary evaluation to distinguish ILA progression from treatment-related lung injury and to guide therapeutic decision-making in this vulnerable population (33).

Genetic susceptibility plays a pivotal role in determining the risk of developing and progressing ILAs (34). Among identified genetic factors, the MUC5B promoter variant (rs35705950) represents the most robust and consistently replicated determinant associated with both ILA presence and fibrotic evolution (34). Carriers of this variant—particularly older individuals, those of European ancestry, and subjects with a family history of pulmonary fibrosis—show a significantly increased likelihood of developing fibrotic patterns and radiologic progression over time (35). Mechanistically, MUC5B overexpression in distal airways impairs mucociliary clearance and promotes aberrant epithelial repair, thereby facilitating fibrotic remodeling (36). Although this variant is strongly linked to disease progression at the population level, its predictive value for individual outcomes remains limited (37). Consequently, current guidelines do not recommend genetic testing for routine monitoring, as follow-up strategies should continue to rely on imaging, clinical symptoms, and pulmonary function assessment (1).

Genetic testing, including the analysis of the MUC5B promoter variant or telomere-related genes (TERT, TERC, RTEL1, PARN) and surfactant-related genes (SFTPA1, SFTPA2, SFTPC, ABCA3), is also not recommended as an initial screening tool (38). Such testing may, however, be considered in patients with a strong family history of pulmonary fibrosis, early-onset disease before 50 years of age, or clinical features suggestive of a telomeropathy (e.g., premature greying, liver disease, or bone marrow dysfunction) (38). In these selected cases, genetic testing may contribute to defining familial risk and guiding counseling of relatives, but it should always be interpreted in conjunction with clinical and radiologic findings, rather than used as a stand-alone screening approach.

ILA are detected in approximately 24–26% of first-degree relatives of patients with familial pulmonary fibrosis, as shown by meta-analyses and large cohort studies (1, 39, 40). This prevalence is substantially higher than that observed in the general population, indicating a significant contribution of genetic and/or shared environmental risk factors. Building on this, longitudinal data demonstrate that among relatives with mild ILAs, approximately 65% progress to more extensive radiologic abnormalities or to clinically overt ILD over a 6-year period, with an even higher risk of progression in those with moderate ILAs (41–44). The clinical significance of ILAs in these high-risk relatives is further underscored by their association with restrictive physiology, impaired gas exchange, and an increased risk of progression to clinically significant ILD and mortality (45, 46). Importantly, early ILAs often represent subclinical disease, and progression rates are markedly higher in individuals with baseline radiologic abnormalities. Indeed, from a prognostic perspective, short telomere length is associated with worse clinical outcomes in patients with IPF and other fibrotic ILDs, and it also negatively affects post–lung transplantation outcomes (47–50). Notably, carriers of heterozygous mutations in telomere-related genes exhibit a more rapid functional decline compared with patients with sporadic IPF (51).

Finally, functional pulmonary assessment, including pulmonary function tests (FVC, Total Lung Capacity, Diffusing Capacity for Carbon Monoxide) and symptom evaluation, is a key component of risk stratification in patients with ILAs (52). Reduced or borderline pulmonary function parameters are recognized as high-risk features for disease progression, warranting closer follow-up and multidisciplinary management (53). Multiple studies have shown that lower FVC values and declining functional measures are associated with increased risk of radiologic progression, accelerated lung function loss, and higher mortality (52, 54).

ILA management is based on risk stratification integrating radiological features (extent, morphology, fibrosis), clinical data (respiratory symptoms, lung function), and genetic background. High-risk individuals (e.g., with fibrotic subpleural pattern, traction bronchiectasis, symptoms, or impaired lung function) should undergo systematic follow-up, including repeat chest CT and pulmonary function testing (1, 20).

Increased serum levels of surfactant protein-D (SP-D), matrix metalloproteinases (MMP-1, MMP-7, MMP-13), resistin, and interleukin-6 (IL-6) have been associated with both the presence and progression of ILA (6, 55, 56). Among these, SP-D is an independent predictor of fibrotic ILA (39). These biomarkers reflect ongoing epithelial injury and remodeling and may precede radiologic or functional progression (57).

Short telomere length is associated with increased risk of progression and mortality in ILA, particularly in individuals with a family history of pulmonary fibrosis or features of telomeropathy (58). Nevertheless, ATS does not recommend routine telomere length measurement in all ILA patients, given its inconsistent predictive value and limited clinical utility, except in selected cases with suggestive clinical features (1). Table 2 provides an overview of the key features that identify patients at higher risk of progression to clinically significant interstitial lung disease.

**Table 2:** Key radiological, clinical, environmental, genetic, and biomolecular risk factors associated with ILA progression.

Domain	Risk factors	Rationale
Radiologic	Subpleural fibrotic pattern; traction bronchiectasis/bronchiolectasis; honeycombing; basal & subpleural predominance; greater extent (≥1% lung or ≥5% of a zone).	Strongly linked to progression and mortality; HR up to 8.4 for fibrotic ILAs.
Clinical/physiology	Age >50; male sex; reduced/borderline FVC, TLC, DLCO; respiratory symptoms (cough, exertional dyspnea).	Older age and impaired function predict radiologic progression, accelerated decline, and mortality.
Environmental	Current/former smoking; dust/fume/mold/air-pollution exposures; occupational risks.	Independent association with fibrotic ILAs, faster decline, and higher mortality.
Genetic	MUC5B promoter variant; short telomeres; family history of pulmonary fibrosis.	Increased risk of presence/progression and worse survival; telomere testing not routine except select cases.
Biomarkers	Elevated SP-D (independent predictor of fibrotic ILA); ↑MMP-1/−7/−13, resistin, IL-6; (selective) KL-6.	Reflect epithelial injury/remodeling; signal higher progression risk; biomarkers not for universal screening.
Comorbidities	CTDs/autoantibodies (e.g., anti-MDA5), telomeropathies, familial pulmonary fibrosis.	Linked to rapidly progressive ILD phenotypes; justify intensified monitoring.
Oncologic context	Active or prior malignancy; exposure to oncologic therapies (chemotherapy, immune checkpoint inhibitors, thoracic radiotherapy).	Associated with higher risk of treatment-related pulmonary toxicity, increased progression to clinically significant ILD, and worse respiratory outcomes; supports individualized risk stratification and closer surveillance.
Familial predisposition	First-degree relative with familial pulmonary fibrosis (FPF) or idiopathic pulmonary fibrosis; presence of ILAs in relatives.	Markedly increased prevalence of ILAs (≈24–26%) compared with the general population; high rates of progression from subclinical ILAs to clinically significant ILD; reflects combined genetic susceptibility and shared environmental exposures; supports targeted screening and closer longitudinal surveillance.

## 4 Monitoring and follow-up strategies

Patients with ILAs who require closer monitoring are those with clinical, radiologic, or comorbid features associated with a higher risk of progression to ILD (Table 2) (1, 2).

According to the 2025 American Thoracic Society guidelines, individuals with newly identified ILAs should undergo a comprehensive initial evaluation (1). This evaluation should involve asking about symptoms such as cough and shortness of breath during exercise, and assessing risk factors. Additionally, lung function tests, including spirometry, lung volumes, and diffusing capacity for carbon monoxide (DLCO), are necessary to establish a baseline for future comparisons (1, 2, 59).

Patients are also advised to stop smoking, limit environmental and occupational exposures, and keep up with their vaccinations (59).

During follow-up visits, symptoms should be monitored regularly. Lung function tests should be repeated every 6–12 months for high-risk patients and every 2–3 years for those at low risk. Chest CT scans should be performed annually or every 2–3 years, depending on the patient’s risk level (1, 2).

Baseline lung biopsy is not recommended for patients with ILAs. In fact, according to the 2025 American Thoracic Society statement, there is no evidence that baseline histopathological analysis improves diagnosis or predicts progression in these individuals, and the potential risks of invasive procedures outweigh the benefits (1). Current guidelines advise against routine biopsies for patients with ILAs since they are a provisional diagnostic category. However, if there is suspicion of a specific interstitial lung disease based on clinical, functional, or imaging findings, histopathological confirmation may needed. Surgical lung biopsy or transbronchial cryobiopsy may be appropriate to establish a definitive diagnosis which is essential for guiding treatment. In the absence of a clear diagnosis, antifibrotic or immunosuppressive therapies are not justified for patients with ILAs.

Even though serum biomarkers, such as KL-6, have prognostic potential, they are not routinely indicated at baseline for all patients with ILAs (60). However, KL-6 testing may be considered in selected cases where there is uncertainty about disease activity or early interstitial involvement, as elevated levels can support the presence of alveolar epithelial injury (60). It may help identify patients at higher risk of progression.

In CTD-ILD, the American College of Rheumatology recommends explicit closer follow-up in patients with worsening symptoms, a UIP pattern, or declining lung function (61).

Prospective cohort studies further demonstrate that even mild or moderate ILAs, particularly among relatives of patients with familial pulmonary fibrosis, determine a substantially increased risk of progression and justify structured monitoring (42).

Multidisciplinary team (MDT) involvement is crucial for the optimal follow-up of patients with ILAs, particularly in risk stratification, monitoring, and management of comorbid conditions (62). The American Thoracic Society recommends that MDTs—typically including pulmonologists, radiologists, rheumatologists, and, when indicated, geneticists—integrate clinical, imaging, physiologic, and genetic data to guide individualized monitoring strategies and management decisions (1). For risk stratification, MDTs assess high-risk features, including family history of pulmonary fibrosis, CTDs, significant inhalational exposures, autoantibodies, and imaging findings (e.g., honeycombing, traction bronchiectasis, subpleural reticulation). This collaborative approach ensures that patients with these risk factors receive appropriately intensified monitoring, including more frequent HRCT and PFTs, as well as timely referral to subspecialists. In monitoring, MDTs coordinate the use of HRCT, PFTs, and, where appropriate, adjunctive biomarkers such as KL-6 and telomere length, whose results must be interpreted in the context of clinical and radiologic findings (1). For comorbidity management, MDTs facilitate comprehensive evaluation of CTDs, familial pulmonary fibrosis, and environmental or occupational exposures, ensuring that rheumatologic, genetic, and occupational aspects are addressed alongside pulmonary care. This integrated model reduces diagnostic delay, improves risk assessment, and supports shared decision-making for individualized patient care.

Discontinuation of monitoring in ILAs may be considered in low-risk individuals who demonstrate prolonged radiologic stability, absence of progression, and lack of high-risk features such as subpleural fibrotic changes or honeycombing (63). Evidence from longitudinal cohort studies indicates that most ILAs remain stable over short-term follow-up (2–3 years), but a significant subset—particularly those with fibrotic features—progress over longer periods, warranting continued surveillance in higher-risk groups (14). It is important to note that modest radiologic changes in individuals with minimal ILAs over 2–3 years often do not reflect significant shifts in symptoms or FVC. The progression to a progressive pulmonary fibrosis (PPF) phenotype is typically gradual and may take years. Thus, frequent follow-ups may overestimate the clinical relevance of minor imaging changes that do not signify meaningful disease progression. The ATS suggests follow-up chest CT at 2–3 year intervals, with shorter intervals for those at increased risk of progression; however, the decision to discontinue monitoring should be individualized, based on radiologic stability, absence of clinical risk factors (older age, male sex, reduced FVC), and lack of radiologic progression (1).

Radiologic progression, especially the development of subpleural fibrosis or honeycombing, is associated with increased mortality and risk of developing interstitial lung disease, supporting ongoing monitoring in these populations (64). Current guidelines and reviews emphasize that there is limited direct evidence or consensus on specific criteria for safely discontinuing surveillance, and most recommendations advocate for individualized assessment rather than routine cessation of monitoring. Further research is needed to establish evidence-based thresholds for stopping follow-up in ILAs (1).

In summary, ILA patients most likely to benefit from structured or intensified follow-up are those with high-risk clinical backgrounds, fibrotic features on CT, abnormal lung function, or comorbidities such as CTDs, familial pulmonary fibrosis, or autoantibody-driven conditions associated with rapid progression. Biomarkers such as KL-6 and telomere length may provide adjunctive prognostic information. Still, current evidence does not support their use as primary monitoring tools or cost-effective alternatives to standard follow-up. HRCT (every 2–3 years, or earlier in high-risk cases), annual PFTs, and MDT-based care remain the cornerstones of ILA surveillance, with biomarker testing considered only in select scenarios and individualized, tailored to the individual patient context.

## 5 “To treat”—arguments in favor of early intervention

Early intervention is crucial for ILAs and early ILD, as these conditions are often irreversible and may follow an unpredictable clinical course, becoming apparent only after prolonged follow-up (65). This creates a tangible risk of missing a therapeutic window to preserve lung function, particularly given that currently available antifibrotic therapies primarily slow—rather than stabilize or reverse—disease progression (66). ILAs have been associated with respiratory symptoms and measurable functional impairment, including reduced pulmonary function test results and lower DLCO, compared with individuals without ILAs (67). Recent evidence further suggests that even modest declines in lung function may carry prognostic significance, underscoring the potential clinical relevance of early functional changes (65, 68).

Nonetheless, assessing and demonstrating treatment benefit in ILAs presents significant challenges for clinicians and researchers. Disease progression is typically slow, heterogeneous, and variably captured across studies, limiting the sensitivity of conventional short-term endpoints. An additional challenge lies in distinguishing ILAs from early ILD, as the commonly used threshold of approximately 5% parenchymal involvement is subjective and may vary across readers, imaging techniques, and assessment methods, particularly in the presence of fibrotic abnormalities (1).

Current statements, recommend against routine treatment of stable, asymptomatic ILAs without high-risk features, but emphasize the importance of early diagnosis and timely management if ILD is suspected or confirmed (1). Therefore, once an ILA diagnosis is established, it is crucial to identify the presence of risk factors for potential progression. ILAs should then treated when there is evidence of progression, development of symptoms and/or decline in lung function, therefore meeting criteria for clinically significant interstitial lung disease (1, 2).

The radiological progression rate towards fibrosing forms ranges from 20 to 76% during medium- to long-term follow-up with a median time to progression of approximately 3–4 years (13, 69).

The antifibrotic agent nintedanib, as demonstrated in the INPULSIS and INBUILD trials, has been approved for the treatment of IPF and other progressive fibrosing interstitial lung diseases (PF-ILDs) (70, 71).

There is evidence in the literature supporting the early initiation of antifibrotic therapy in fibrosing lung disease. In fact, data extrapolated from the placebo group of the INPULSIS trial showed that the rate of FVC decline over 1 year, compared to baseline, was similar in patients with preserved lung function (FVC > 90%) and those with more impaired FVC at baseline (72). Moreover, a post-hoc analysis of pooled data from the INPULSIS trials demonstrated that the effect of nintedanib in reducing the annual rate of FVC decline was comparable in patients with GAP stage I (Gender, Age, Physiology) and those with more advanced stages (GAP II and III) (73, 74).

In a study conducted by Sugino et al. (75), it was observed that in patients with early IPF (defined as Stage I by the Japanese Respiratory Society scoring system), both nintedanib and pirfenidone led to a smaller decline in FVC % pred over 6 months compared to placebo. The greatest benefit was observed in patients exhibiting desaturation during the 6-min walk test (6MWT) or with higher GAP stages (II/III). It is important to recognize that interstitial lung disease may be the initial or the only manifestation of an underlying systemic autoimmune disorder. In such instances, early treatment can be warranted even before systemic symptoms appear, as prompt intervention may alter the disease’s progression and prevent irreversible lung damage. Consequently, the decision to begin treatment should not be based solely on a diagnosis of early IPF. Instead, it should consider the possibility of an autoimmune or genetic background, along with the overall risk of disease progression, assessed through clinical, functional, and radiological evaluations.

Given the fibrotic progression rate of interstitial lung diseases associated with telomere-related gene mutations, Justet et al. (76) evaluated the efficacy and tolerability of nintedanib and pirfenidone in this patient cohort, highlighting a reduction in FVC decline. Importantly, when a short telomere syndrome is identified or even suspected, antifibrotic therapy should be promptly initiated, as these patients typically exhibit a high risk of rapid progression (77). Moreover, telomere-related interstitial lung diseases encompass a broader spectrum beyond idiopathic pulmonary fibrosis, including other fibrosing phenotypes with similar clinical behavior and therapeutic implications (78). These findings suggest the potential efficacy of antifibrotic therapy in early-stage IPF and in genetic forms of ILD related to telomeropathies (76, 79). Therefore, nintedanib and pirfenidone might be considered in high-risk ILA patients as a preventive strategy against the development of pulmonary fibrosis (80). However, prospective studies are needed to confirm this hypothesis. In addition to currently approved antifibrotic agents, emerging therapies may further expand future treatment options for fibrotic lung disease (81, 82). Nerandomilast, a novel antifibrotic agent, has recently demonstrated efficacy in patients with IPF and PPF, mainly in combination with nintedanib. Co-administration with pirfenidone has been evaluated in IPF, where the 18 mg dose of nerandomilast showed efficacy, whereas this combination has not yet been studied in patients with PPF (81, 82). Importantly, clinical trials suggest a favorable tolerability profile, which may represent a relevant advantage for long-term treatment strategies (83). Although data on nerandomilast in interstitial lung abnormalities are currently lacking, such agents may be particularly attractive for future investigation in early or high-risk ILA populations. The main criteria supporting an early therapeutic intervention are summarised in Table 3.

**Table 3:** Main advantages and disadvantages of early treatment in patients with interstitial lung abnormalities (ILAs).

Domain	Arguments “to treat”	Arguments “not to treat”
Clinical rationale	Prevent progression to clinically significant ILD/fibrosis in high-risk cases.	Natural history is heterogeneous; many patients are asymptomatic and may never progress.
Evidence base	Antifibrotics (nintedanib, pirfenidone) slow FVC decline in IPF and PF-ILD; benefit seen even with preserved FVC.	No trials specifically in early, asymptomatic ILAs; benefits extrapolated from other ILDs.
Timing	Earlier initiation may capture a window to modify fibrotic trajectory.	“Watchful waiting” avoids overtreating stable/non-progressive ILAs.
Safety	Adverse effects often manageable with dose adjustments/supportive care.	Frequent GI side effects, hepatotoxicity, photosensitivity; adherence may suffer in asymptomatic patients.
Economics/QoL	Potential to avert downstream costs of advanced disease (inference from PF-ILD data)	Very high annual drug costs for antifibrotic (> $100 k) with unproven benefit in ILAs; therapy can raise anxiety and impair QoL.
Guidelines raccomandation	Treat when there is progression, symptoms, lung function decline, or criteria for clinically significant ILD.	Routine treatment of stable, asymptomatic ILAs is not recommended; structured surveillance is.

## 6 “Not to treat”—arguments for watchful waiting

Not all ILDs require active treatment. Except for IPF—for which antifibrotic therapy is recommended in all patients—longstanding and clinically stable ILDs often do not benefit from therapeutic intervention, especially in the absence of radiologic or functional progression (84, 85). Patients with normal pulmonary function tests or with minimal, stable abnormalities are typically classified as having clinically significant ILD, for which the appropriate management is careful observation and regular follow-up rather than pharmacological therapy (86). A more debatable situation arises when the DLCO is reduced, but no respiratory symptoms or radiologic progression occur. In these cases, treatment decisions should be individualised—balancing the risk of overtreatment and drug-related adverse effects against the potential benefit of early intervention to prevent future decline (69). Evidence currently available regarding the management of ILA focuses on the correct timing of radiological, functional, and clinical follow-up.

In fact, even if antifibrotic therapy is approved for the treatment of PPF, including the ILA phenotype, no data are currently available regarding its efficacy in early ILAs, which are characterized by an uncertain and variable rate of progression (85). Recent studies indicate that the clinical impact of antifibrotic therapies varies by disease stage, with greater absolute benefit observed when treatment is initiated earlier in the disease course. This variation does not imply a reduction in therapeutic efficacy over time; instead, it reflects the progressive nature of fibrotic ILD and the influence of baseline disease severity on treatment outcomes (71). Although antifibrotic agents have demonstrated sustained efficacy in reducing the rate of lung function decline in idiopathic pulmonary fibrosis and other progressive fibrosing interstitial lung diseases, they do not completely arrest disease progression. Evidence from randomized trials and real-world studies indicates that the relative treatment effect is preserved over time, whereas the absolute clinical benefit may appear attenuated in advanced disease stages or after substantial functional decline (87). These observations underscore the importance of treatment timing and support a watchful waiting strategy in stable or asymptomatic ILAs, with therapy initiation reserved for cases demonstrating clear radiologic or functional progression (14).

Another not negligible critical aspect is the safety profile of antifibrotic agents. In patients treated with antifibrotics, the most common side effects include gastrointestinal symptoms, such as nausea, diarrhoea, dyspepsia and weight loss (88). Diarrhoea is the most frequent side effect in patients treated with nintedanib (88). It occurs in about 60% of patients, while nausea is the most common in patients taking pirfenidone (about 30% of patients), followed by photosensitizing reactions (15%). Both drugs can cause hepatotoxicity, especially nintedanib (13 vs. 2.9%) (89, 90). These side effects can be managed with symptomatic treatment or dose reduction, but often lead to drug discontinuation. However, antifibrotics represent only one component of the therapeutic approach to ILD, which also includes the management of comorbidities, pulmonary rehabilitation, oxygen therapy, and, in selected cases, lung transplantation (53). Considering the burden of treatment-related adverse events and the limited impact of current therapies on disease course, maintaining an acceptable quality of life often becomes as relevant as prolonging survival. Initiating an antifibrotic therapy in asymptomatic patients with ILAs could, therefore, expose them to several side effects without any proven therapeutic benefit. Moreover, patients with ILAs are generally asymptomatic; therefore, treatment compliance may be suboptimal in the absence of perceivable clinical symptoms. On the other hand, therapy initiation might cause anxiety about the condition and potentially lead to deterioration in quality of life.

Although immunosuppressive therapy is not routinely indicated for the management of ILAs, these findings should not be interpreted as uniformly unrelated to conditions that may later require immunomodulatory treatment (91). According to the Fleischner Society and recent reviews, management should focus on clinical and radiologic surveillance rather than pharmacologic intervention (2). Immunosuppressive therapy is reserved for ILDs with a clear inflammatory or autoimmune basis—such as connective tissue disease–associated ILD, hypersensitivity pneumonitis, or sarcoidosis—where corticosteroids, azathioprine, mycophenolate mofetil, rituximab, or tocilizumab may be used according to disease-specific evidence (53). In contrast, in fibrotic ILDs such as idiopathic pulmonary fibrosis, immunosuppression is ineffective or even harmful (85). The use of immunosuppressive drugs, particularly in older adults, carries significant risks, including opportunistic infections, cytopenias, hepatotoxicity, nephrotoxicity, metabolic disturbances, osteoporosis, and increased cardiovascular and malignancy risk (92). Corticosteroids, even at low doses and with long-term use, are associated with hyperglycemia, hypertension, bone fragility, and neuropsychiatric effects, with a particularly high risk of serious complications in frail or comorbid patients (93). For these reasons, in patients with ILAs lacking clear evidence of inflammatory or autoimmune activity, initiating immunosuppression would expose individuals—particularly the elderly—to potentially significant adverse effects without proven benefit. A careful follow-up strategy remains essential to identify those who progress to clinically meaningful ILD and may subsequently benefit from appropriate therapeutic intervention.

Additionally, these drugs carry a significant economic burden, with an annual cost of therapy—either with pirfenidone or nintedanib—generally above $100,000 per patient (94, 95). This expense may not be accompanied by an equal benefit in terms of reduced hospitalisations, resource utilisation, or improvements in quality of life—especially considering the uncertain natural history of ILAs (96).

The Official American Thoracic Society Clinical Statement regarding ILAs suggested intervening in risk factors for progression, such as smoking cessation, reducing exposure (e.g., environmental, occupational, medication), and vaccinations1.

In the absence of definitive evidence supporting early pharmacologic intervention in ILAs and considering the heterogeneity of disease trajectories, management decisions should integrate patient preferences and shared decision-making principles. These discussions should evaluate the potential benefits and risks of early treatment compared to structured surveillance, address the uncertainty of disease progression, and consider the possible impact on quality of life, especially in individuals who are largely asymptomatic.

In conclusion, treating ILAs may lead to adverse effects and increased utilization of healthcare resources without any demonstrated clinical or economic benefit. The main criteria supporting a conservative follow-up strategy are summarized in Table 3 Therefore, a structured clinical, radiological, and functional follow-up is essential to identify patients who exhibit disease progression as early as possible and to ensure timely and appropriate therapeutic intervention only when it is needed.

## 7 Conclusions and future directions

ILAs are an increasingly recognized radiologic finding, representing a stage along the continuum between normal lung ageing and clinically significant ILD (97, 98). Although frequently asymptomatic and incidentally discovered, ILAs—particularly those with subpleural fibrotic features—are linked to a higher risk of progression to pulmonary fibrosis, functional decline, and increased mortality (99). The heterogeneous natural history of ILAs underscores the need for precise risk stratification, which involves integrating radiologic, functional, clinical, and genetic factors to inform individualized management.

Future research should focus on prospective, multicenter studies aimed at establishing validated criteria for disease progression, defining optimal surveillance strategies, and identifying therapeutic thresholds. Randomized clinical trials assessing antifibrotic agents or other disease-modifying interventions in high-risk ILA populations are essential to determine whether early pharmacologic treatment can modify the disease trajectory (97). A concise overview of the proposed clinical, radiological, and functional criteria to guide follow-up and management decisions in ILAs is summarized in Table 4.

**Table 4:** Recommended monitoring and follow-up strategy for patients with ILAs according to current evidence and ATS 2025 guidance.

Domain	What to do	When/Who	Notes
Baseline assessment	Evaluate respiratory symptoms Exposures PFT HRCT	At first ILA identification (all patients).	Establish reference for change; classify pattern (non-subpleural vs. subpleural; fibrotic vs. non-fibrotic).
Risk stratification	Integrate CT features, PFTs, symptoms, age/genetics/comorbidities; MDT review.	Early after baseline in suspected higher risk.	
Lifestyle/prevention	Smoking cessation; minimize environmental/occupational exposures; vaccinations up to date.	All patients.	Core risk-reduction measures for everyone with ILA.
Routine PFT follow-up	High-risk: every 6–12 months. Low-risk: every 2–3 years.	—	—
Imaging follow-up	High-risk: HRCT annually Low-risk: HRCT every 2–3 yo	—	—
Biomarkers/genetics (selective)	Consider KL-6 if diagnostic/prognostic uncertainty; consider telomere length or gene panels (TERT/TERC/MUC5B) with strong FHx/early onset/telomeropathy signs.	Selected patients only.	—
Biopsy	Not recommended at baseline in ILAs.	—	No evidence biopsy improves diagnosis or predicts progression.
Escalation triggers	New/worsening symptoms; ≥5–10% relative FVC fall or DLCO drop; increased fibrosis extent or new traction/honeycombing on HRCT.	Prompt MDT review	Movement from ILA to clinically significant ILD merits treatment consideration.

In conclusion, ILAs provide a critical window for early recognition of individuals at risk for pulmonary fibrosis (1). A multidisciplinary, evidence-based, and risk-adapted approach remains central to management (1). Continued advances in imaging technologies, molecular profiling, and genetic analysis are expected to enhance prognostic precision and foster the development of targeted preventive strategies in this rapidly evolving field.
